# Repair of full-thickness articular cartilage defects using IEIK13 self-assembling peptide hydrogel in a non-human primate model

**DOI:** 10.1038/s41598-021-83208-x

**Published:** 2021-02-25

**Authors:** Alexandre Dufour, Jérôme E. Lafont, Marie Buffier, Michaël Verset, Angéline Cohendet, Hugues Contamin, Joachim Confais, Sharanya Sankar, Marika Rioult, Emeline Perrier-Groult, Frédéric Mallein-Gerin

**Affiliations:** 1 Laboratory of Tissue Biology and Therapeutic Engineering, CNRS UMR 5305, University Claude Bernard-Lyon 1 and University of Lyon, 7 Passage du Vercors, 69367 Lyon Cedex 07, France; 2 3-D Matrix Europe SAS, Caluire, France; 3 Cynbiose, Marcy l’Etoile, Lyon, France

**Keywords:** Osteoarthritis, Regenerative medicine

## Abstract

Articular cartilage is built by chondrocytes which become less active with age. This declining function of the chondrocytes, together with the avascular nature of the cartilage, impedes the spontaneous healing of chondral injuries. These lesions can progress to more serious degenerative articular conditions as in the case of osteoarthritis. As no efficient cure for cartilage lesions exist yet, cartilage tissue engineering has emerged as a promising method aiming at repairing joint defects and restoring articular function. In the present work, we investigated if a new self-assembling peptide (referred as IEIK13), combined with articular chondrocytes treated with a chondrogenic cocktail (BMP-2, insulin and T3, designated BIT) could be efficient to restore full-thickness cartilage defects induced in the femoral condyles of a non-human primate model, the cynomolgus monkey. First, *in vitro* molecular studies indicated that IEIK13 was efficient to support production of cartilage by monkey articular chondrocytes treated with BIT. *In vivo*, cartilage implant integration was monitored non-invasively by contrast-enhanced micro-computed tomography, and then by post-mortem histological analysis and immunohistochemical staining of the condyles collected 3 months post-implantation. Our results revealed that the full-thickness cartilage injuries treated with either IEIK13 implants loaded with or devoid of chondrocytes showed similar cartilage-characteristic regeneration. This pilot study demonstrates that IEIK13 can be used as a valuable scaffold to support the *in vitro* activity of articular chondrocytes and the repair of articular cartilage defects, when implanted alone or with chondrocytes.

## Introduction

Articular cartilage is built by only one type of cells, the chondrocytes, which become less active with age and injury. This declining function of the chondrocytes, together with the avascular nature of the cartilage, impedes the spontaneous recovery of full-thickness defects. If left untreated, these lesions can progress to more serious degenerative articular conditions, ultimately reaching the subchondral bone compartment as in the case of osteoarthritis (OA). The knee joint is particularly affected by traumas and OA, representing a major cause of disability worldwide^1,2^. OA treatments are mainly symptomatic (e.g. paracetamol, non steroidal anti-inflammatory drugs, NSAIDs) and none of them offer any relevant effect for structural regeneration or stabilization of the cartilage matrix^3^. Total knee arthroplasty (TKA) is a last resort solution for treating knee joint OA but the young population faces the limited lifespan of knee prostheses. Early OA can also generate from a focal lesion of cartilage and knee injury is known to be a strong risk factor for OA^4^. The surgical procedures currently used to treat focal articular defects do not restore full articular function and have a high complication rate in the long term. For instance, microfracture often leads to the production of fibrocartilage^5^ and mosaicplasty suffers from a lack of integration between host and donor cartilage^6^. Therefore, finding new therapeutic options enabling complete healing of cartilage or osteochondral defects still constitutes a challenging unmet clinical need in the field of OA prevention.

In this context, cartilage tissue engineering approaches have emerged with the aim to repair joint defects and restore articular function, thus eliminating or at least delaying the need for joint replacement. Among these approaches, matrix-induced autologous chondrocyte implantation (MACI) has been established as an attractive and hopeful therapy^7^. Such minimally invasive therapy combines the use of chondrocytes and biomaterials but requires a substantial number of chondrocytes that lose their phenotype during *in vitro* amplification, by a phenomenon called dedifferentiation^8^. During this process, chondrocytes stop expressing specific markers such as type II collagen in favor of the fibrotic type I collagen^9^. Consequently, the risk of reconstructing fibrocartilage with different biomechanical and physiological properties compared to the hyaline articular cartilage is non-negligible. Two major parameters are important to optimize MACI: soluble factors for controlling the chondrocyte phenotype and biomaterials to provide support for tissue growth. On the one hand, the choice of soluble factors relies on the basic knowledge of chondrocyte differentiation. On the other hand, the rationale of using biomaterials as scaffolds for tissue regeneration is to obtain a temporary three-dimensional (3D) structure for the *in vitro* growth of live cells and their subsequent implantation into the lesion area. In previous studies, we investigated the *in vitro* reconstruction of the cartilaginous matrix by human articular chondrocytes (HACs) seeded in collagen sponges. We used soluble factors during HAC expansion (fibroblast growth factor (FGF)-2 and insulin, designated FI) and culture in collagen sponges (bone morphogenetic protein (BMP)-2, insulin and triiodothyronine T3, designated BIT), a combination that was originally optimized for human auricular chondrocytes in collagen gels^10^. We found that the FI-BIT combination allowed high HAC proliferation and cartilage-characteristic extracellular matrix (ECM) production in collagen sponges^11–13^. However, the risk that implantation of animal-derived collagen biomaterials could trigger adverse immune reactions encouraged us to explore the use of alternative synthetic scaffolds.

Hydrogels are widely accepted as biomaterials for cartilage tissue engineering. Concerning chondrocyte physiology, hydrogels present advantageous internal structures in comparison with collagen sponges. For instance, chondrocytes tend to stretch out in the porous structures of the collagen sponges, as in monolayer culture^14^, which promotes actin polymerization and chondrocyte de-differentiation^15^. Instead, hydrogels induce round cellular morphology and actin depolymerization, a cytoskeletal organization favoring chondrogenic expression^16^. Among hydrogels, self-assembling peptides (SAPs) represent a new class of hydrogels for tissue engineering. These synthetic peptides consist of short peptide sequences and studies have shown that they do not elicit an immunogenic or inflammatory response *in vitro*^17^ or *in vivo*^18^. SAPs are advantageous for articular cartilage repair since the self-assembling process results in highly hydrated material (more than 90% water content)^19^. This is consistent with articular cartilage being highly hydrated (60–80% water content)^20^, a feature offering the tissue its "shock absorber" capabilities. Their self-assembling feature also makes them well suited for injection directly into non-uniform cartilage defects^21^. Very recently, we used a new SAP, referred to as IEIK13, and we showed that the combination of IEIK13 and human nasal chondrocytes treated with BIT can form a cartilage gel *in vitro*^22^. With the background of the *in vitro* studies, in the present work, we further aim to investigate if the combination of IEIK13 hydrogel with BIT-treated HACs could be effective as a potential injectable hydrogel to treat articular cartilage defects.

We sought to test the safety and efficacy of IEIK13 to repair full-thickness cartilage defects induced in the femoral condyles of a non-human primate model, the cynomolgus monkey (*Macaca fascicularis*). Studies using large animal models are necessary to verify the functionality of reconstructed cartilage *in vivo*. Quadrupeds are often used to test the repair of cartilage defects, but most quadrupeds show several angulations in the bone alignment of hind limbs, resulting in highly flexible joints that makes it difficult to extrapolate the results to human physiology. Unlike them, non-human primates show the bone alignment of hind limbs similar to humans, which is not unrelated to the opportunistic use of bipedal gait in some species, as is the case for cynomolgus monkey^23^. Also, the distal femora in monkeys and humans present similarities in their anatomical contours^24^. The cartilage repair process was assessed in the cynomolgus macaque using a non-invasive imaging technique: the computed tomography (CT). Although magnetic resonance imaging (MRI) has been successfully used to assess cartilage repair *in vivo*^25–27^, CT is a more readily available and cost-effective imaging technique^28^. Micro-CT is mainly used to perform qualitative and quantitative assessments of bony structures in animal studies owing to the high contrast between calcified and soft tissues^28–31^. However, the use of radiopaque contrast agents opens the possibility to assess the morphology and biochemical composition of soft tissues such as cartilage^32^ but, to our knowledge, this technique has never been used to monitor implantation of a cartilage construct *in vivo*.

In summary, we tested the hypothesis that the combination of IEIK13 SAP and BIT-treated articular chondrocytes would be efficient to restore cartilage defects in the cynomolgus monkey. Since there were no previous studies using monkey articular chondrocytes (MACs) for cartilage repair, we first confirmed the potential of MACs to be responsive to the same FI and BIT concentrations that we used for amplification and redifferentiation of HACs in our previous studies^11–13^. We performed real-time PCR and Western-blotting analyses to compare gene expression and protein production of MACs cultured in IEIK13 and fibrin, a biopolymeric material commonly used in orthopaedic surgery as a fixative and more recently proposed as a cell carrier for MACI^33–35^. After implantation of IEIK13- and fibrin-based cartilage gels in a cynomolgus model of cartilage injury, we performed non-invasive monitoring of implant stability using contrast-enhanced micro-computed tomography (CECT). Three months post-implantation, the treated articular surfaces were collected post-mortem and analysed by histological and immunohistochemistry staining.

## Results

### MACs and HACs show similar *in vitro* redifferentiation capacities in fibrin and IEIK hydrogels

**Figure 1:**
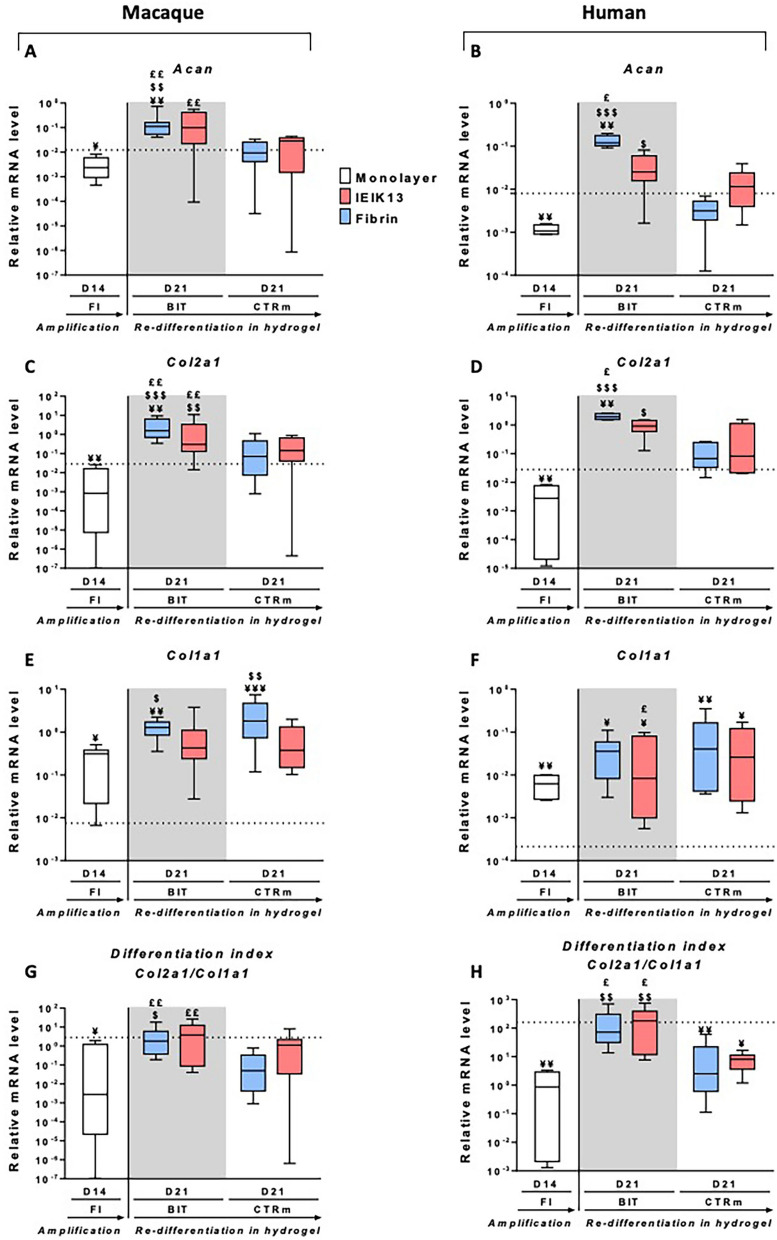
Effect of culture conditions on the mRNA steady-state levels of specific markers, as indicated, in macaque and human chondrocytes. After extraction, chondrocytes were amplified on plastic for 14 days (D14) in the presence of FGF-2 and insulin (FI). Then, they were seeded in fibrin or IEIK13 hydrogel and cultivated for 21 days (D21) in control medium (CTRm) or in medium containing BMP-2, insulin and T3 (BIT). Data are presented as box plots (median, quartiles, extreme values). The horizontal dotted lines correspond to mRNA levels of chondrocytes 48 h after their isolation time (values reported as medians). (**A**,**B**) Relative mRNA expression of *Acan*. (**C**,**D**) Relative mRNA expression of *Col2a1*. (**E**,**F**) Relative mRNA expression of *Col1a1*. (G,H) *Col2a1:Col1a1* mRNA ratio. The results shown were obtained with chondrocytes isolated from 6 human donors and 9 animals. (¥: significant effects vs. 48 h chondrocytes, as determined using the Mann–Whitney test; $: significant effects vs. D14 chondrocytes amplified with FI, as determined using the Kruskal–Wallis test; £: significant effects vs. D21 chondrocytes cultivated in control medium, as determined using the Wilcoxon test; ¥, $, £, *p* < 0.05; ¥¥, $$, ££, *p* < 0.01; ¥¥¥, $$$, £££, *p* < 0.001).

**Figure 2:**
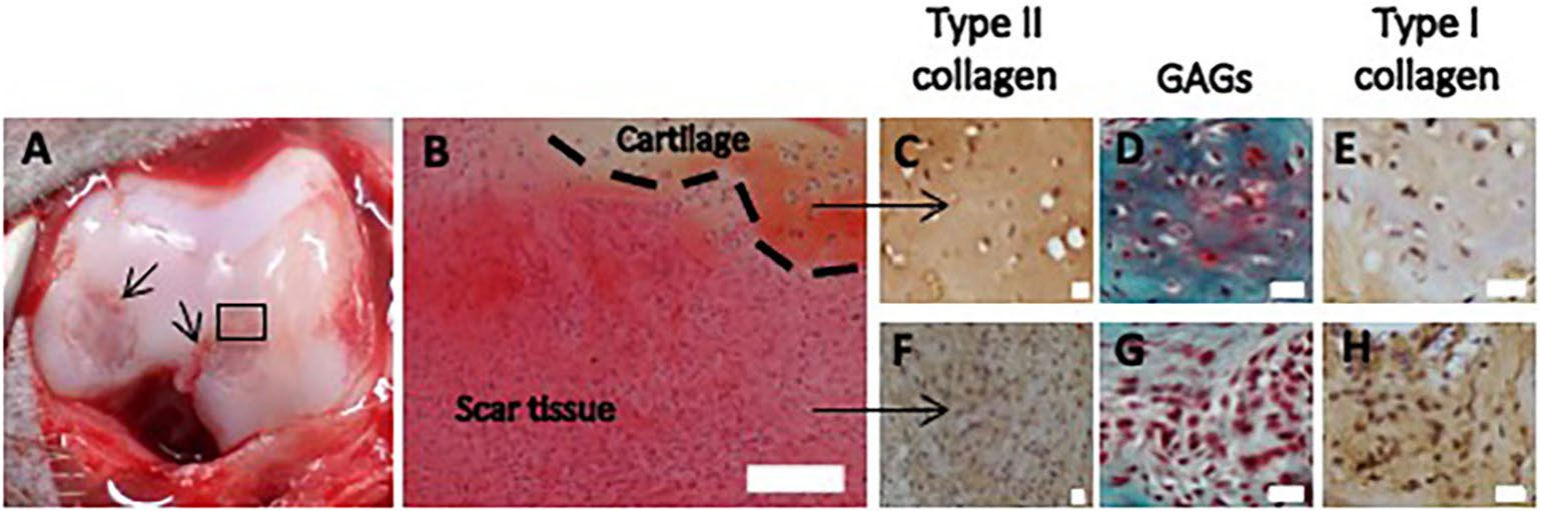
Representative macroscopic image (**A**) and histological sections (**B**–**H**) of scar tissue and native cartilage observed at day 0, before debridement of the defects. (**A**) The small arrows indicate the edges of the defects. The square refers to the histological section in (**B**). (**B**) Hematoxylin and eosin staining of the border of the defect. The dotted line indicates the interface zone between scar tissue and native cartilage (scale bar: 200 μm). (**C**–**E**) and (**F**–**H**) depict detailed regions of cartilage and scar tissue, respectively, with staining of GAGs and immunostaining of type I and type II collagen, as indicated (scale bar: 20 μm).

MACs and HACs were cultivated for 2 weeks on plastic in the presence of FI and then for 3 weeks in fibrin or IEIK13 hydrogel in the presence or absence of BIT. First, gene expression analysis was performed to quantify the relative abundance of the transcripts coding for type II collagen (*Col2a1)* and aggrecan (*Acan*), two major components of cartilage. Similar profiles of gene expression were obtained for MACs and HACs: *Col2a1* and *Acan* expressions were lost after expansion in monolayer and regained after culture in fibrin or IEIK13 hydrogel (Fig. 1A–D). More precisely, the BIT cocktail promoted the re-expression of *Col2a1* and *Acan* to higher levels in comparison with the control medium (Fig. 1A–D). Of note, the stimulatory effect of BIT was particularly significant for MACs, revealing good reactivity of this animal cell model to the chondrogenic cocktail (Fig. 1A,C). We also investigated *Col1a1*, the gene coding for the α1 chain of type I procollagen, a classical marker of chondrocyte dedifferentiation. *Col1a1* expression increased following cell amplification, then remained stable or underwent small variations during culture in fibrin or IEIK13 hydrogel (Fig. 2E,F). To better apprehend the status of the chondrocyte phenotype, we calculated the functional differentiation index corresponding to the ratio of *Col2a1* mRNA to *Col1a1* mRNA levels. For both MACs and HACs, the ratios reached the highest values in the presence of BIT, without significant differences between fibrin and IEIK13 (Fig. 1G,H). More precisely, the values obtained in the presence of BIT were in the same range as the values measured in P0-48 h chondrocytes, which is a sign of good restoration of the chondrocyte phenotype (Fig. 1G,H). In complement, western-blot analysis of type I and type II collagen synthesized by MACs and HACs showed that BIT provoked robust production of type II collagen in fibrin- and IEIK13-based cartilage substitutes, whereas type I collagen synthesis was not detected or detected to a lesser extent (see Supplementary Figure S1). Taken together, these data indicated that MACs were as responsive as HACs to the chondrogenic BIT cocktail and IEIK13 was as efficient as fibrin to support articular chondrocyte redifferentiation and cartilage matrix production.

### Intra-articular implantation of fibrin- and IEIK13-based cartilage constructs in monkey

We further investigated the feasibility of using SAP IEIK13 cartilage gels to repair articular cartilage defects, in comparison with fibrin-based cartilage gels. Full-thickness articular defects were created in femoral condyles of adult monkeys and were left as is until the day of implantation of the cartilage substitutes. This 5-week time period allowed the formation of a scar tissue that was removed and analyzed by immunohistochemistry for type I and type II collagen and by Safranin-O staining to reveal the presence of glycosaminoglycans (GAGs) of sulfated proteoglycans. The reparative tissue covering the cartilage defect was strongly positive for type I collagen and weakly positive for type II collagen and GAGs, suggesting that the repair tissue was a fibrotic tissue. This was in contrast with the native cartilage present at the border of the biopsy which appeared barely positive for type I collagen and clearly positive for type II collagen and GAGs (Fig. 2).

**Figure 3:**
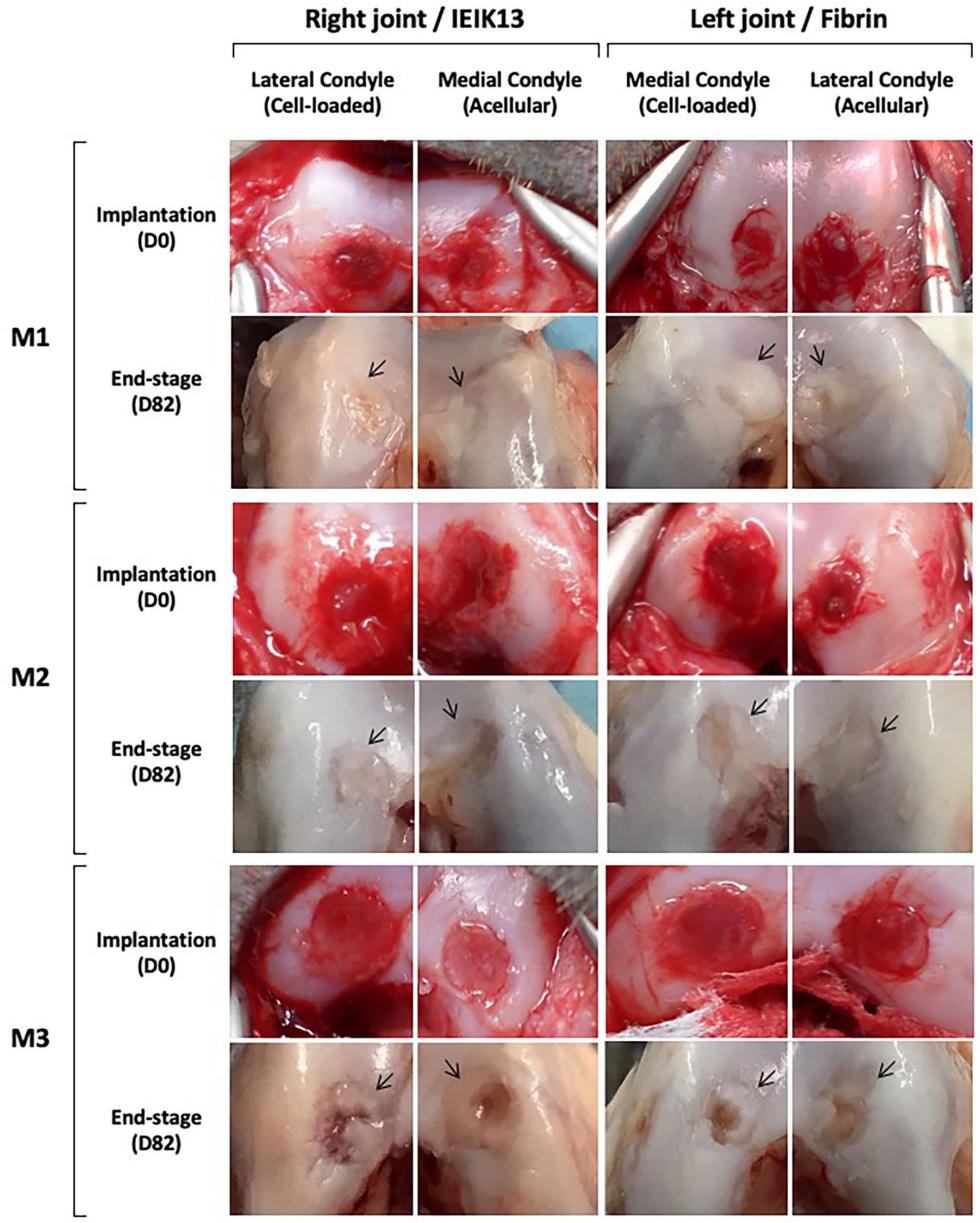
Macroscopic observations of the cartilage defects filled by IEIK13- and fibrin-based hydrogels at the day of implantation (D0) and 82 days after implantation (D82). The hydrogels were loaded or not with chondrocytes, as indicated. The anatomical site of implantation in the knee joint is specified and M1, M2 and M3 correspond to 3 *Cynomolgus* monkeys. The arrows indicate the original defect margin. The M1 and M2 defects appear globally well resurfaced whereas the central regions of M3 defects appear depressed or filled with rough tissue.

**Figure 4:**
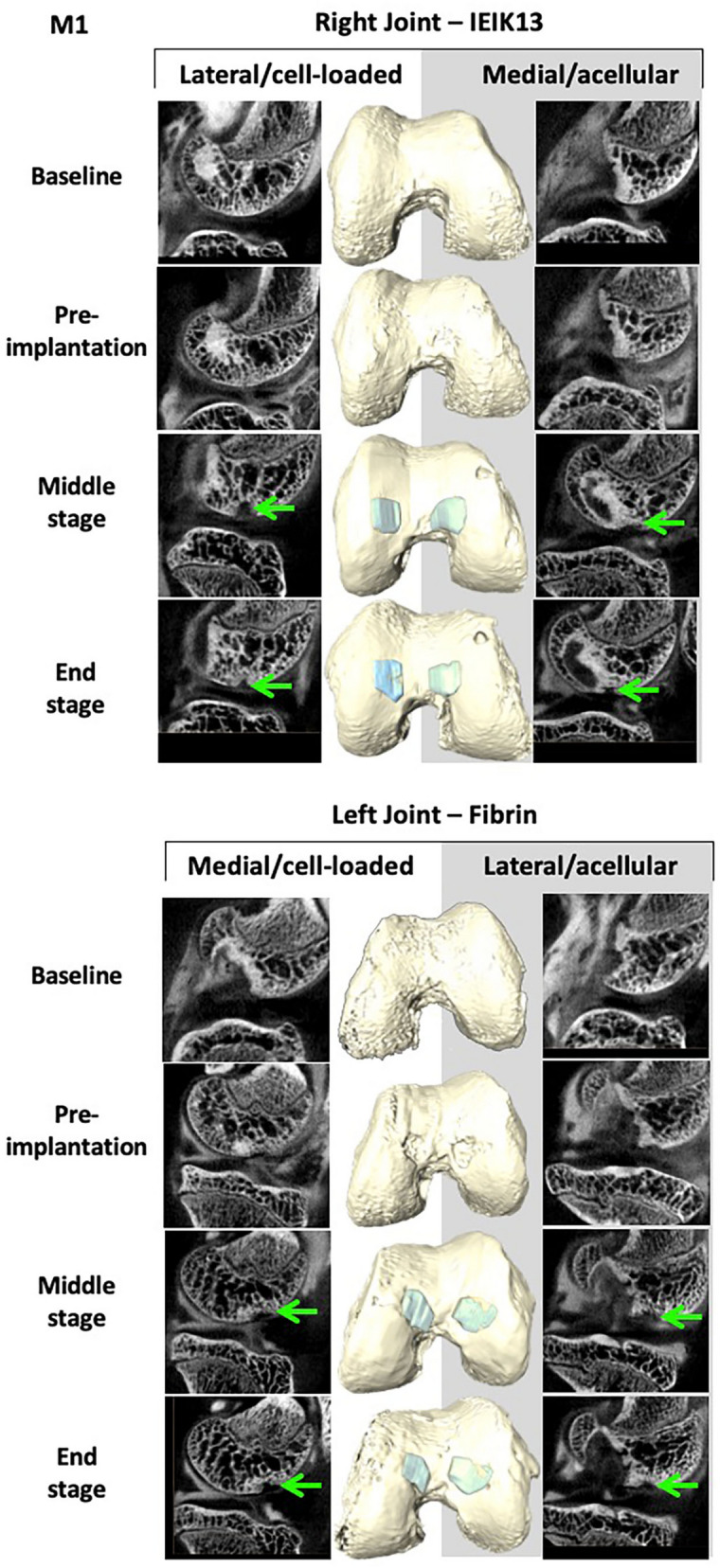
Non-invasive monitoring of IEIK13- and fibrin-based hydrogels implanted in M1 monkey. The hydrogels were loaded or not with cells, as indicated. The site of implantation in knee joint is specified. Examples of segmented CECT images (left and right panels) and 3D surface reconstruction (middle panel) corresponding to different stages of the study: 3 days before first surgery (baseline), 6 days before removal of the scar tissue and implantation (pre-implantation), 41 days after implantation (middle-stage) and 82 days after implantation (end-stage). The green arrows show absence of contrast agent, indicating occupancy of the corresponding regions by the implants.

**Figure 5:**
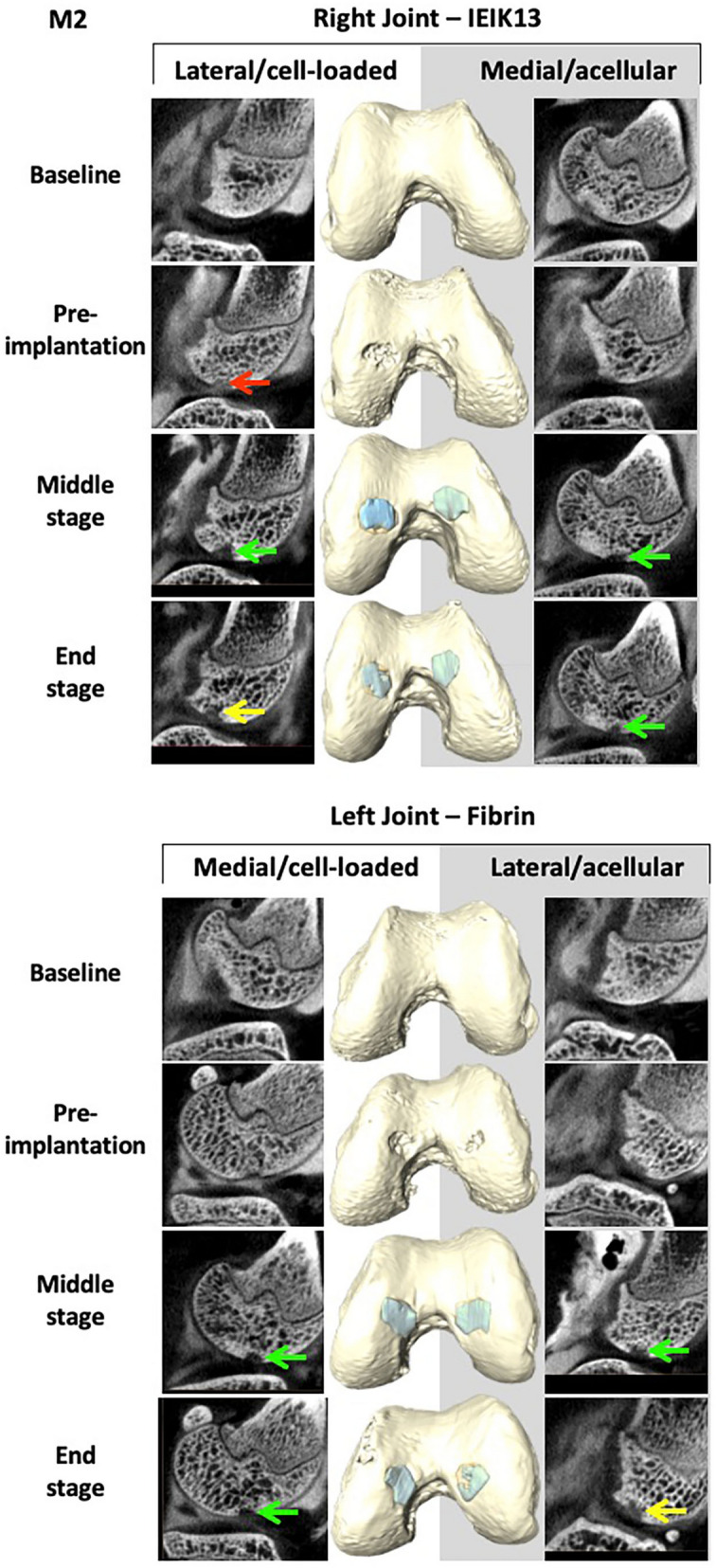
Non-invasive monitoring of IEIK13- and fibrin-based hydrogels implanted in M2 monkey. The hydrogels were loaded or not with cells, as indicated. The site of implantation in knee joint is specified. Examples of segmented CECT images (left and right panels) and 3D surface reconstruction (middle panel) corresponding to different stages of the study: 3 days before first surgery (baseline), 6 days before removal of the scar tissue and implantation (pre-implantation), 41 days after implantation (middle-stage) and 82 days after implantation (end-stage). The red arrow shows complete filling of the defect by the contrast agent (observed as grey cloud). The yellow arrows show heterogeneous diffusion of the contrast agent. The green arrows show absence of contrast agent, indicating occupancy of the corresponding regions by the implants.

**Figure 6:**
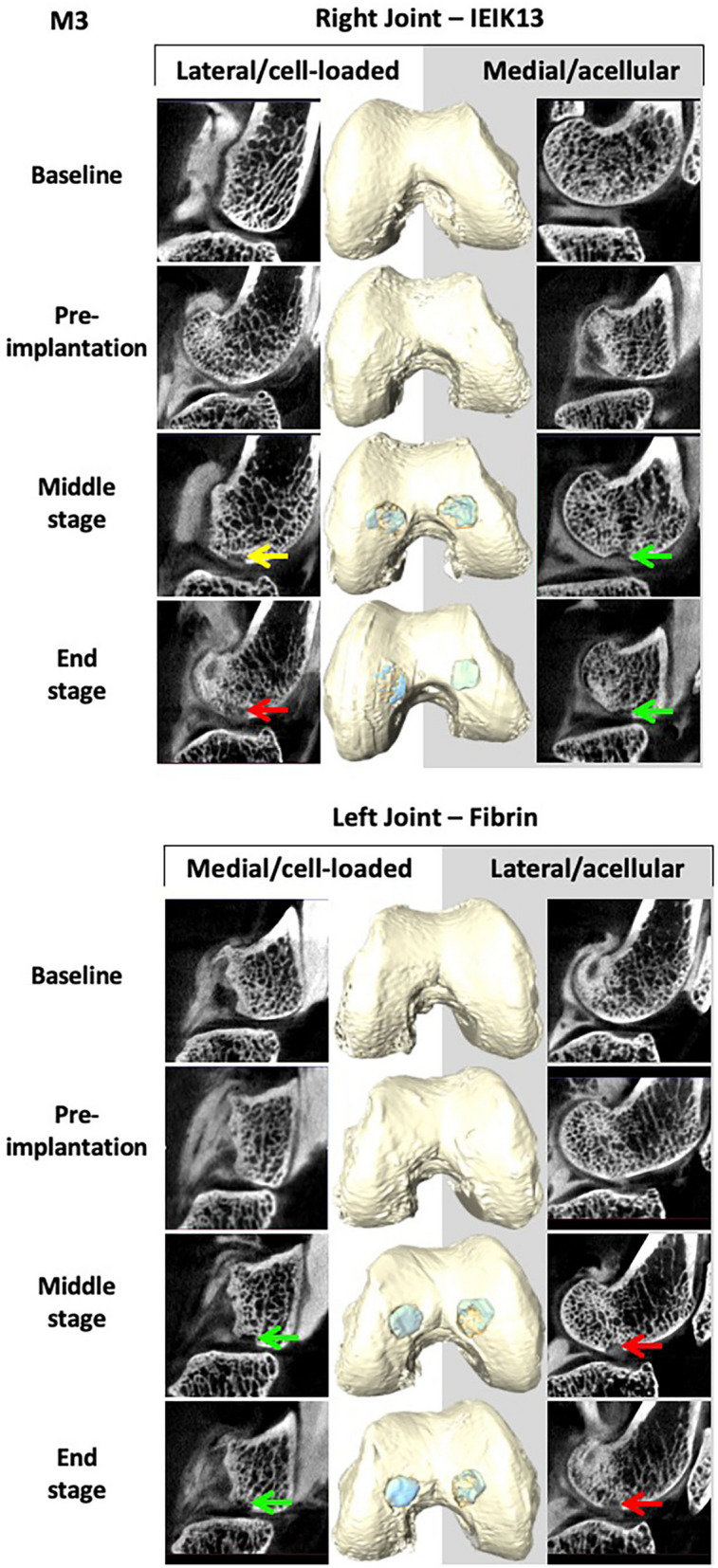
Non-invasive monitoring of IEIK13- and fibrin-based hydrogels implanted in M3 monkey. The hydrogels were loaded or not with cells, as indicated. The site of implantation in knee joint is specified. Examples of segmented CECT images (left and right panels) and 3D surface reconstruction (middle panel) corresponding to different stages of the study: 3 days before first surgery (baseline), 6 days before removal of the scar tissue and implantation (pre-implantation), 41 days after implantation (middle-stage) and 82 days after implantation (end-stage). The red arrows show complete filling of the defects by the contrast agent (observed as grey cloud) and the yellow arrow shows heterogeneous diffusion of the contrast agent. The green arrows show absence of contrast agent, indicating occupancy of the corresponding regions by the implants.

Following surgical debridement of the cartilage defects, the IEIK13- and fibrin-based hydrogels, with or without chondrocytes, were implanted. Macroscopic observations demonstrated that the cartilage defects were fully covered by all the implants on the day of implantation (day 0) (Fig. 3). No sign of pain or distress was observed in 3 monkeys (designed M1, M2 and M3) following the first and the second surgery. The animals were capable of bearing weight and moved normally throughout the study. The monkeys were euthanized after 82 days and the implant and cartilage integration was evaluated macroscopically. First, general observations showed resurfacing of all defects for M1 and M2 whereas uncomplete resurfacing was observed for the defects induced in M3 (Fig. 3). Further, the quality of the repair process could be macroscopically scored according to the International Cartilage Repair Society (ICRS) score system^36^, as follows: grade II (= nearly normal cartilage) for the M1 and M2 defects and grade III (= abnormal cartilage) for the M3 defects (Fig. 3). The healing process of the 3 animals was monitored non-invasively by CECT and a 3D surface rendering of the joints was built from 2D section images. The joints were imaged at different stages of the study: 3 days before first surgery (referred to as baseline), 6 days before implantation (referred to as pre-implantation), 41 days after implantation (referred to as middle-stage) and 82 days after implantation (referred to as end-stage). The 3D surface rendering predicted that all the cartilage injuries of M1 and M2 were globally well filled by the implants, at the middle- and end-stage of the study (Figs. 4 and 5). Concerning M3, only one defect appeared correctly resurfaced (by the fibrin cell-loaded implant) at the end-stage of the study whereas the other defects appeared incompletely filled. This lack of filling was already observed at the middle-stage of the study (Fig. 6).

**Figure 7:**
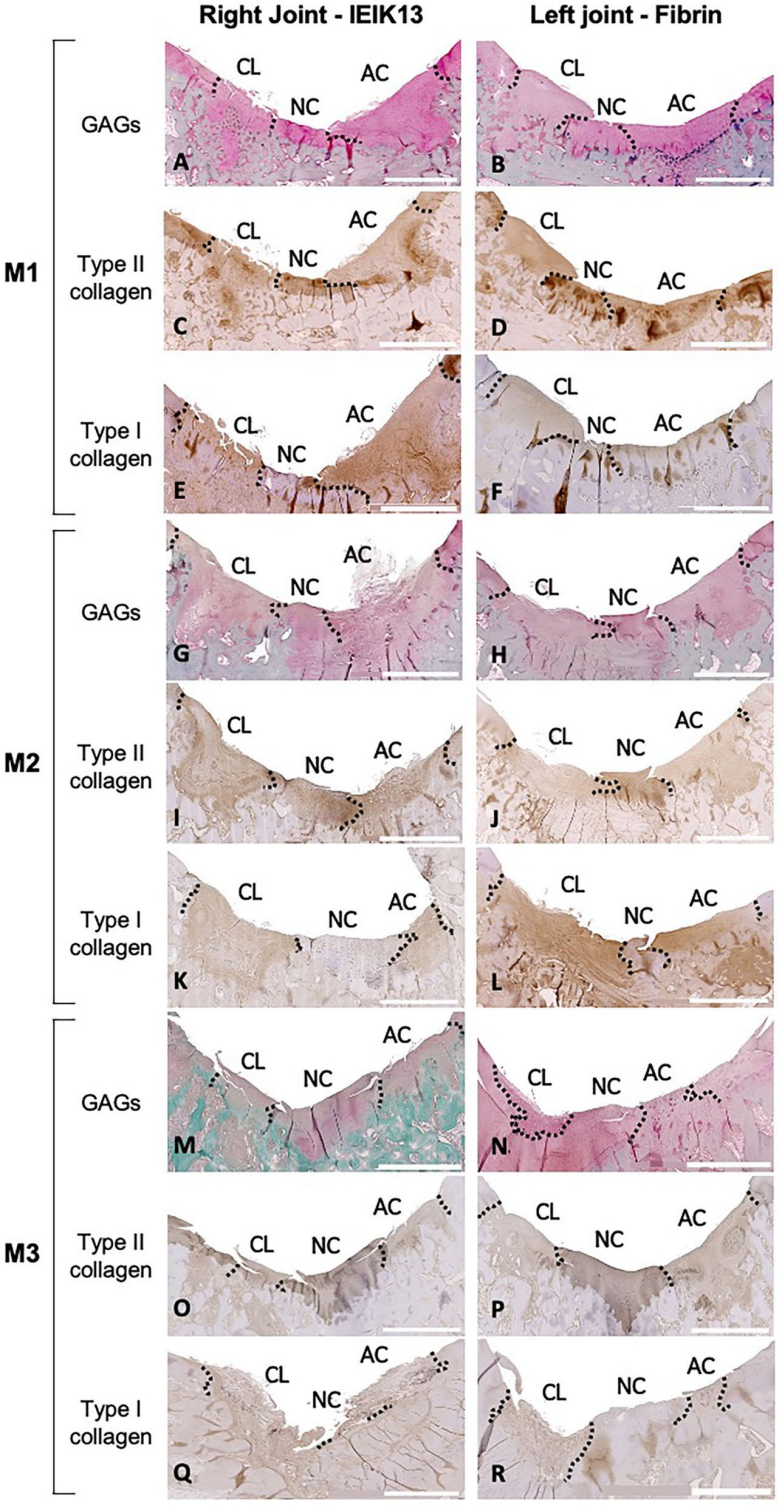
Histological characterization of the reparative tissues in femoral condyles of M1, M2 and M3 monkeys, as indicated. GAG staining and type I and type II collagen immunostaining are shown on coronal sections. The hydrogel type and the anatomical site of implantation are indicated (*CL* Cell-loaded, *NC* Native Cartilage, *AC* Acellular, scale bar: 2 mm). The boundaries between the implants and the surrounding cartilage are represented by dotted lines.

The articular surfaces were further analyzed histologically, and tissue sections were processed for GAG staining and type I and type II collagen immunostaining. The results are presented in Fig. 7 and histological images of normal articular cartilage explanted from a non-operated joint are presented as a reference in Supplementary Figure S2. The boundaries between the implants and the surrounding cartilage were visible on all the frontal sections but the quality of resurfacing was variable, from smooth surface (Fig. 7B,D,F,P,K,N) to fibrillated surface (Fig. 7A,C,E,G,I,L,Q,R) with occasional fissures (Fig. 7H,J,M,O). The regenerated tissues stained for GAGs and type II collagen, demonstrating the cartilage nature of the neo-synthesized matrix although these stainings appeared somewhat less intense than those observed in the native cartilage region. In parallel, the regenerated tissues stained weakly for type I collagen whereas type I collagen was absent in native cartilage and present in the underlying bone structures (Fig. 7A–R). Interestingly, the tissues treated with IEIK13 and fibrin implants showed similar intensities of staining for GAGs and collagens, whether or not the implants were originally loaded with chondrocytes (Fig. 7A–R). These findings suggested that implants were colonized by host cells capable of chondrogenic differentiation. In support of this, a detailed examination of the sections revealed that all the implants were cellularized, even the implants originally devoid of chondrocytes (Fig. 7 and Supplementary Figure S3). Furthermore, the implants appeared to be protruding into the underlying bone (Fig. 7A–R), which must have facilitated the invasion of bone marrow-mesenchymal stem cells (BM-MSCs) into the implant. Indeed, we observed that the cells found in these protrusions stained with antibodies against CD56 and CD146 (Supplementary Figure S3), two cell-surface markers typical of MSCs^37^.

## Discussion

This work represents the first animal study aiming to determine if IEIK13 SAP is a suitable scaffold biomaterial to repair full-thickness articular cartilage defects. Since our approach was to use MACI, we compared this novel synthetic hydrogel with fibrin gel that was already used clinically for MACI^33^. We demonstrated that MACs can be redifferentiated to produce cartilaginous matrix with equivalent efficacy in IEIK13 and fibrin, using the same dose of BIT cocktail as for HACs. The *in vitro* data hence provided a convincing basis to continue the studies with IEIK13 as a scaffold to repair articular cartilage defects created *in vivo* in a cynomolgus monkey model.

As demonstrated in our results, spontaneous healing took place in 3.5-mm defects and resulted in fibrous tissue. However, Araki et al.^38^ showed that spontaneous healing occurred only with defects up to 2 mm diameter in the *Cynomolgus* macaque. In that study however, the defects were created in the groove of the femoral trochlea which is a non-weight bearing area in comparison with the lateral and medial condyles that were used in our study design. Thus, the mechanical constraints must have caused the spontaneous healing of the defects in spite of the defect size being larger than 2 mm. Supporting this idea and in keeping with our observations, one of the other few studies using non-human primates for cartilage repair research reported that 3 mm diameter defects created in the femoral condyles and left untreated for 24 weeks led to the formation of fibrous tissue^39^. Therefore, although we did not leave the defects untreated for the whole period of our study, we believe that the scar tissue removed at 5 weeks would not have evolved further into hyaline-like cartilage.

Very interestingly, the 3D surface rendering of the joints at the end-stage of the study was in good concordance with our macroscopic observations. The overall good quality of the resurfacing predicted by 3D imaging for the defects of animals M1 and M2 was clearly confirmed during the autopsy by the presence of a rather white hyaline cartilage-like tissue at their surface. The incomplete filling of the 4 M3 defects, as judged by the brownish color observed macroscopically in the central regions, was also predicted by 3D imaging even though the 3D surface rendering showed the defect of the left medial condyle to be filled to a larger extent than was observed macroscopically. In the latter case, it is still possible that the center of the defect was filled up to the level of the native cartilage since it is difficult to appreciate retrospectively from the photographs if the brownish color corresponds necessarily to a hollow-like deformity. A recent study has demonstrated the use of micro-CT 2D images to build a 3D surface rendering of the joints for treatment of osteochondral defects with tissue engineering methods, but the imaging was performed ex vivo, following the explantation of their femoral condyles^31^. To our knowledge, our study is the first to show 3D surface rendering of cartilage grafts on live animals. We have successfully demonstrated the efficacy of this method to non-invasively investigate the cartilage implant integration in an experimental model that is very close to humans, particularly in mimicking the native biomechanics of the joints. Since modern micro-CT devices have circular openings with diameters large enough for *in vivo* CECT imaging of a large animal joint, we think that this method, more readily available and cost-effective than MRI, could be developed for longitudinal monitoring of tissue-engineered cartilage implants *in vivo*. Interestingly, multidetector CT-arthrography (MDCTA) represents an important clinical technique that is now commonly applied to patients unable to stand an MRI examination^40^. This suggests that CECT-based 3D imaging may be used to survey postoperatively tissue-engineered cartilage implants in human patients. However, it should be kept in mind that this technique assesses the filling of a defect, not the quality of the reconstructed cartilage matrix.

Adhesion and lateral integration of a tissue-engineered cartilage with the surrounding native tissue is crucial for its function in the context of joints^41^. The histological analysis showed that all the implants anchored well to the subchondral bone and adjacent native cartilage. These results are thus consistent with the macroscopic observation of the cartilage and 3D surface imaging of M1 and M2 joints, but not for M3 joints: in contrast to histological observations, three out of four M3 defects appeared incompletely filled according to macroscopic observation and 3D reconstruction. In fact, when assessing experimental cartilage defect repair, correlations between macroscopy, imaging techniques and histology are usually hard to find and depend on quantitative scoring systems^42^. Here for example, histological analysis was based on a limited number of tissue sections and cannot be spatially representative of implant integration. However, histological observations show information that is not revealed by macroscopy or 3D surface imaging: the surfaces of IEIK13 and fibrin implants, loaded with chondrocytes or not, showed fibrillation or irregularities on certain sections. This was true not only for M3 but also for M1 and M2, whose repaired defects showed macroscopic smooth surfaces. These findings altogether are globally in line with the general view that good correlations between macroscopic and histological scoring systems exist mainly for structural categories such as "defect fill" and "integration" rather than for the categories like "surface"^42^.

The initial goal of the present study was to explore the use of a new therapeutic implant containing chondrocytes to repair articular defects limited to cartilage. However, because of the limited thickness of the articular cartilage in the cynomolgus monkey (about 1 mm), it was technically difficult to restrict the creation of articular defects to the superficial layer of cartilage. Indeed, all observations confirm that the subchondral bone was exposed at the base of the defects (bleeding seen at the defect sites, CECT and histological sections showing defects reaching the bone). The lack of difference between the cartilage injuries repaired with implants loaded with or devoid of chondrocytes can be explained by the "marrow-stimulating" character of our approach. This raises the interesting possibility that IEIK13 hydrogel could be used more simply as an acellular scaffold to repair full-thickness articular cartilage. A very recent study using another SAP (RADA16-I) functionalized with a BM homing peptide to repair full-thickness articular cartilage defects in rabbit has demonstrated increased recruitment of endogenous BM-MSCs and chondrogenic differentiation, in comparison with classical microfracture^30^. In the work presented here, IEIK13 was not intended to attract MSCs but our results suggest that IEIK13 was as effective as fibrin to recruit BM-MSCs. This is well supported by the identification of CD56 and CD146 positive cells in the zone of implant extruding through the underlying bone. Regarding fibrin, our results are also in agreement with a previous study using an equine model where full-thickness cartilage lesions treated with MSC-seeded fibrin or fibrin alone showed similar histological appearance, with equivalent GAG, type I and type II collagen biochemical contents, suggesting that fibrin allows the migration of BM-MSCs capable of chondrogenic differentiation^43^. In our study, it remains difficult to say if cell homing acted as an adjunctive for the implants containing chondrocytes since the relative stainings of type II collagen and GAGs (two chondrocytic markers) and of type I collagen (a marker of both dedifferentiated chondrocytes and MSCs) were of the same intensity in all the implants. It is possible that the duration of the study (≈ 12 weeks) may have been too short to catch significant differences in the quality of repair between the experimental conditions. It is also possible that another study design with larger defects (e.g. 5 mm in diameter) could result in a more clear-cut difference in the degree of cartilage repair between the implants containing chondrocytes or not.

Overall, the current study established that IEIK13 SAP is a suitable scaffold for the redifferentiation of amplified articular chondrocytes and for the repair of full-thickness articular cartilage defects. Most importantly, we found an equivalent performance for the novel IEIK13 hydrogel compared to fibrin, already tested as gel carrier in MACI^33^. In comparison with fibrin, IEIK13 has the advantage of presenting low risks of biological pathogen transmission and allows homogeneous production batches. Besides, we found that IEIK13 implants were well accepted by the monkeys and did not cause any histological sign of an inflammatory reaction, which is in agreement with previous studies showing that SAPs do not elicit an immunogenic or inflammatory response *in vitro*^17^ or *in vivo*^18,44^. Thus, this pilot study, using the biomechanically closest animal model to humans, demonstrated for the first time that IEIK13 acts as a valuable scaffold for articular cartilage repair. We did not find superior capacity of chondrocyte-loaded IEIK13 over acellular IEIK13 to repair cartilage defects. Longer studies (6–12 months) including a larger number of animals are now required to assess tissue maturation over time and to consider the need for cell delivery in combination with IEIK13. Very interestingly, the use of IEIK13 without the need for cell transplantation would offer great benefits for future clinical development.

## Material and methods

### Animal study design

**Figure 8:**
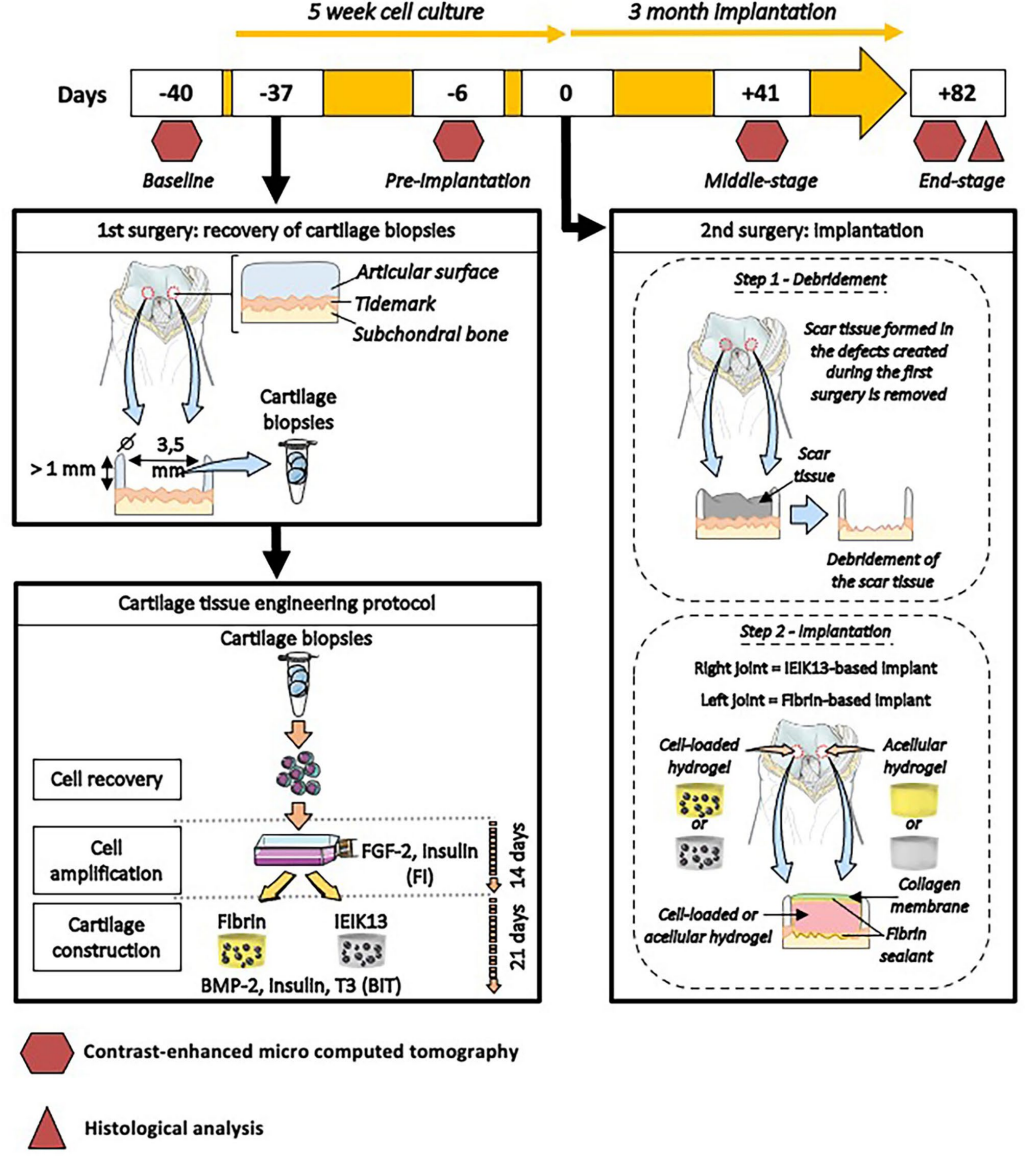
Experimental design of the animal study showing the preparation process of the cartilage constructs made of IEIK13 or fibrin gel combined to chondrocytes. The key times of the study are referred as numbers of days before or after the day of implantation (day 0).

The animal study was reviewed by the Animal Welfare Body of Cynbiose and the Ethics Committee of VetAgro-Sup (Marcy l’Etoile, France) and was approved by the French Ministry of Higher Education and Research under number 1716V2 (MESR No. 201703811141169). The animal protocols and experiments were performed in compliance with the ARRIVE guidelines and in accordance with the official regulation on animal experimentation (European Directive 2010/63/EU and its national transposition). The testing and animal housing facility (Cynbiose, Marcy-l’Etoile, France) has full accreditation from the Association for Assessment and Accreditation of Laboratory Animal Care (AAALAC International). Three skeletally mature female cynomolgus macaques (*Macaca fascicularis*), approximately 3.6, 5.6 and 6.7 years old and weighing 3.6, 3.5 and 3.3 kg, respectively, were used. An overview of the experimental procedure pursued during our study is described in Fig. 8.

A first surgery was performed to collect articular cartilage biopsies for isolation and culture of autologous chondrocytes. Frustoconical biopsies were delineated on distal medial and lateral femoral condyles of both knees of each animal with a 3.5 mm diameter biopsy punch, then collected with a scalpel. After 5 weeks (corresponding to the time necessary for chondrocyte cultivation), a second surgery was performed and the same zones were again delineated with a 3.5 mm diameter biopsy punch and debrided. The defects created were then gently dug with a bone curette until the subchondral bone was exposed. Then, IEIK13 and fibrin hydrogels, loaded or not with MACs, were implanted in the defects by using fibrin sealant (TISSEEL, Baxter). The anatomical sites of implantation of the different hydrogel types and loadouts are specified in Table 1. To secure the implants, a collagen membrane (Viscofan BioEngineering) was glued on the top of the hydrogels with fibrin sealant. Three months after implantation, animals were killed by an intravenous injection of a euthanizing agent following general anesthesia and knee joints were collected for histological analysis.

**Table 1:** Distribution of hydrogel implants according to anatomical sites of the joints. The table is representative of two knees of the same animal.

Right knee—IEIK13 implants		Left knee—fibrin implants	
Lateral femoral condyle	Medial femoral condyle	Medial femoral condyle	Lateral femoral condyle
Hydrogel + chondrocytes	Acellular hydrogel	Hydrogel + chondrocytes	Acellular hydrogel

### Animal welfare

The animals were socially housed in a large enclosure, on natural bedding. The housing was ventilated with more than 8 air changes/h, with controlled temperature (22 ± 4 °C) and humidity (55 ± 20%) and with a 12-h light/dark photoperiod. Litter maintenance was performed daily. Litter replacement, animal room and enclosure full cleaning were performed at least once a month. Animals were fed with a standard diet (100 g/animal/day) and were provided daily with fresh fruits or vegetables and unsweetened treats. As part of the testing facility environmental enrichment program, the animals had continuous access to toys and foraging devices. Tap water was available *ad libitum* through a water bottle with a sipper tube and was changed at least once a day. Body weight was measured once a week from the beginning to the end of the study. All animals were monitored at least once a day for food consumption, the appearance of clinical signs and assessment of pain and distress throughout the study.

### Surgical procedures and post-operative recovery

Surgical procedures were performed under aseptic conditions with repeated sterile irrigation of the articular surface. Animals were fasted at least 8 h before anesthesia. The animals were anesthetized by intramuscular injection of 10 mg/kg ketamine hydrochloride and 0.5 mg/kg midazolam, intravenous infusion of Ringer Lactate through a cephalic venous catheter, endotracheal intubation and maintained with a volatile anesthetic (1% isoflurane). Strong analgesia was provided by intramuscular injection of fentanyl (10 μg/kg at the induction of anaesthesia and post-anaesthesia recovery, then twice a day during 3 post-operative days) and of ketoprofen (2 mg/kg at the induction of anaesthesia and once a day during 5 days post-operatively). Physiological parameters were monitored during the whole anaesthesia with a multi-parameter monitor.

In the initial surgery (collection of cartilage biopsies), a medial arthrotomy followed by lateral patellar luxation was performed to access the surgical site. In the second surgical procedure (implantation of hydrogel-based constructs), a lateral arthrotomy followed by medial patellar luxation was performed to access the surgical site, to avoid re-opening the medial wound. Following intervention on the condyles, a layered closure of the surgical site was performed (joint capsule and medial or lateral fascia, subcutaneous tissue, and skin).

After post-anaesthetic recovery, animals were allowed to move freely in their enclosure and were immediately bearing weight on both lower limbs. Surgical wounds were monitored once a day for 6 days after surgery. The skin staples used for cutaneous closure of surgical sites were removed after 10 post-operative days. Body temperature was measured the day before and the day of surgery, and for 3 days following surgery.

### Non-invasive monitoring of implants by contrast-enhanced micro-computed tomography (CECT)

For the longitudinal analysis of knee repair, macaques were examined at several time points of the study via CECT. The joints of each animal were first examined 40 days before implantation to obtain reference images of healthy joints (referred to as baseline) and, after the creation of the defects, 6 days before implantation to obtain reference images of empty defects (referred to as pre-implantation). The joints were then imaged 41 days and 82 days after implantation (referred to as middle-stage and end-stage, respectively). Imaging sessions were performed with the same general anesthesia conditions as for surgeries. A contrast agent (VISIPAQUE) was diluted to 2:3 in Phosphate Buffer Saline (PBS) and 1 mL of the solution was injected in the synovial cavity of the joints. The lower limbs were then subjected to 10 flexion/extensions to homogenize the contrast solution within the synovial cavity. The joints were rotated inward in the micro x-ray scanner to expose medial and lateral condyles. A SKYSCAN 1278 micro x-ray scanner (Bruker) was used and one acquisition was performed per animal and per time point, imaging both knees at the same time.

Three-dimensional surface rendering was obtained from a series of 2D micro-CT images using AVIZO v4.2.0 software (Thermo Fisher Scientific, USA). Images were used to evaluate the defect and implant volumes and to calculate the percentage of defect volume filled by the implant. For a given defect, an acquisition corresponds to about 50 to 70 images. Manual pre-segmentation was performed around the defect to define a Region Of Interest (ROI) and isolate the corresponding voxels. The ROI was determined in this manner for every 10 images. An interpolation procedure was then used to assign a ROI to every single image, allowing the delineation of the defect’s contours. The defect’s volume was calculated based on those ROIs. A threshold of gray values was then applied to the ROIs to accurately identify voxels corresponding to hydrogel-based implants. A threshold of 799 Hounsfield Unit (HU) was determined based on the Otsu method^45^. Accordingly, voxels with a gray value below 799 HU correspond to hydrogel-based implants and voxels over 799 HU correspond to bone or space filled with contrast agent.

### Human sample collection

HACs were isolated from macroscopically healthy zones of osteoarthritic knee joints obtained from 6 donors (age range 61–77, 50% male and 50% female) undergoing total knee replacement. The study was performed in full compliance with local ethics guidelines, national and European Union legislation regarding human sample collection, manipulation and personal data protection. The study protocol was approved by the ethics committee of COnservation D'ELéments du COrps Humain (CODECOH: DC-2014–2325) for preservation and research with human samples. The cartilage samples were collected after written informed consent of the donors.

### Macaque sample collection

For the studies conducted *in vitro*, MACs were isolated from healthy joints of 9 macaques (age range 4–7, 55% male, 45% female) obtained from Cynbiose and euthanized at the end of another study not related to osteoarticular physiology.

### Isolation and expansion of chondrocytes

HACs and MACs were extracted as previously described^46^. Briefly, small slices of cartilage were digested in a culture medium consisting of Dulbecco’s modified Eagle medium/Ham’s F12 (Gibco Invitrogen) with 0.06% bacterial collagenase A (Roche Applied Science) overnight. The cells were then seeded at a density of 1.5 × 10^4^ cells/cm^2^ on culture dishes with culture medium supplemented with 10% fetal bovine serum (FBS) (Gibco), 100 mg/mL streptomycin and 100 U/mL penicillin (Invitrogen). Forty-eight hours after seeding, the medium was refreshed and further supplemented with 5 ng/mL FGF-2 (R&D Systems) and 5 µg/mL insulin (Umuline Rapide, Lilly), namely the FI cocktail. The culture medium was then replaced three times a week. At confluence, cells were trypsinized, counted with a hemocytometer and used for hydrogel encapsulation.

### Chondrocyte redifferentiation in hydrogel

Trypsinized HACs or MACs were embedded in fibrin or IEIK13 hydrogels. For the preparation of fibrin-based gels, human fibrinogen (Millipore) was dissolved in 10 mM HEPES (Sigma) at pH 7.4 to obtain a 50 mg/mL human fibrinogen solution and human thrombin (Millipore) was dissolved in 10 mM HEPES at pH 6.5 supplemented with 0.1% BSA to obtain a 2 U/mL thrombin solution. A sodium–calcium chloride solution was prepared with 3 M NaCl and 0.4 M CaCl_2_ in 10 mM HEPES at pH 7.4. The cell-fibrin suspension was obtained by mixing 1 × 10^6^ cells resuspended in 275 µl DMEM/F-12 with 100 µL fibrinogen, 100 µL thrombin and 25 µL NaCl/CaCl2 solutions. Volumes of 100 mL of this mixture were distributed into wells of 24-well plates and chondrocyte-fibrin constructs were allowed to gel at 37 °C. After gelling, 1 mL of culture medium containing 1 X ITS (insulin, transferrin and selenium, Gibco), 50 µg/mL 2-phospho-L-ascorbic acid (trisodium salt, Fluka) supplemented or not with 200 ng/mL BMP-2 (Dibotermine-α, drug form of BMP-2 contained in InductOs kit, Wyeth), 5 µg/mL insulin (Umulin, Lilly) and 100 nM T3 (tri-iodothyronine, Sigma) was added to the wells. The combination of BMP-2, insulin and T3 is referred to as BIT. The culture medium containing only 1 X ITS and ascorbate corresponds to the control medium (CTRm). The medium was replaced every 2 days over a culture period of 21 days.

For the preparation of IEIK13-based gels, cells were re-suspended in 10% wt/vol sucrose solution then mixed with a volume of IEIK13 peptide solution prepared as previously described^22^, so that the final peptide solution was 1% (wt/vol) and the cell density was 2 × 10^6^ cells/mL. This mixture was homogenized by reverse pipetting and volumes of 100 µL were distributed into wells of 24-well plates. The wells were then supplemented with 1 mL of DMEM-F12 medium and chondrocyte-IEIK13 constructs were left at 37 °C for 1 h to achieve self-assembling. Then, half of the culture medium was substituted by the control medium or medium containing BIT. For each well, half of the medium was replaced in the same way every 2 days over a culture period of 21 days.

### Preparation of autologous implants

For the *in vivo* study, autologous chondrocytes were isolated from cartilage biopsies collected on 3 macaques during the first surgery (Fig. 1). The cells were extracted and expanded for 14 days in the presence of FI. The cells were then trypsinized, mixed with fibrin or IEIK13 peptide solution and induced to redifferentiate with BIT. The culture conditions were the same as for the *in vitro* redifferentiation assays, except for the following modifications: for preparation of fibrin-based gels, sterilized molten agarose (2.5%) was first poured into wells of 6-well plates and allowed to solidify. In each well, the agarose layer was punched with a 8 mm diameter biopsy punch and the agarose plug was removed. Then, 40.2 µL of fibrin/cell mixture were poured in the punched area to form disc 0.8 mm thick discs. After fibrin gelation, the cell-fibrin constructs were cultivated for 21 days in the presence of BIT. At the time of implantation, the constructs were punched with a 3.5 mm diameter biopsy punch and were implanted into the defects by gentle pressure. For the preparation of IEIK13-based gels, volumes of 300 µL of mixture of cell-IEIK13 peptide solutions were prepared and poured in wells of 24-well plates. After a 21-day culture period in presence of BIT and at the time of implantation, the constructs were collected with a spatula to fill the defects.

### Western-blot analysis

After 21 days of *in vitro* culture, the culture medium was removed from the wells and 100 µL of fibrin- or IEIK13-based constructs were harvested and processed for western-blot analysis as previously described^22^. In brief, the samples were re-suspended and boiled in 300 µL 2 X Laemmli buffer containing 3% β-mercaptoethanol. Twenty (20) µL of samples were separated using sodium dodecyl sulfate–polyacrylamide gel electrophoresis on 4–15% Mini-PROTEAN TGX gradient gels (Biorad). Following the transfer, membranes were probed with primary antibodies, washed and incubated with secondary antibodies (see Supplementary Table S1). After several washes, bound antibodies were detected on X-ray films with Immun-star alkaline phosphatase or horseradish peroxidase (HRP) chemiluminescent substrate (Bio-Rad). When re-probed with antibodies, membranes were first stripped with ReBlot Plus Strong solution (Millipore).

### Histological and immunohistochemical analysis

Macaque femoral condyles were first fixed in formol acetic alcohol (AFA, Microm Microtech) for 2 weeks then decalcified with Rapid Bone Decalcifier (Eurobio) for 10 h. Following decalcification, joints were fixed again in AFA for 4 days, then dehydrated and embedded in paraffin.

Staining and immunostaining were performed on 5 µm coronal sections. Paraffin was removed with methylcyclohexane and sections were rehydrated then incubated with 800 U/mL type IS hyaluronidase (Sigma) for 30 min at room temperature (RT). Endogenous peroxidases were blocked using Bloxall kit (VECTOR Laboratories) for 10 min and unspecific sites were saturated with BlockAid blocking solution (ThermoFisher Scientific) for 20 min at RT or with 2.5% horse serum for 1 h at RT. Sections were then incubated with primary antibodies, followed by incubation with HRP-conjugated secondary antibodies (Supplementary Table S1). Finally, sections were revealed with diaminobenzidine and counterstained with hematoxylin and eosin. To detect sulfated glycosaminoglycans (GAGs) carried by proteoglycans, sections were stained with Safranin-O in 0.1 M sodium acetate (pH 7.4) for 10 min. Image acquisitions were performed with an Axio Scan.Z1 (Zeiss) slide scanner and treatment was performed with the Zen 2.5 software (Zeiss).

### Gene expression analysis by reverse transcription-polymerase chain reaction

Total RNA was isolated from chondrocytes cultivated in fibrin and IEIK13 hydrogels for 21 days, by using the Nucleospin RNA II kit (Macherey–Nagel) according to the manufacturers instructions. Reverse transcription-polymerase chain reaction was performed as previously described^22^. Briefly, reverse transcription was performed using 50 ng total RNA with PRIMESCRIPT RT reagent kit (Takara). Real-time polymerase chain reaction amplification was performed in a 20 μL mix containing 10 μL FastStart Universal SYBR Green Master (Roche), 4 μL cDNA (1:3 dilution), 300 nM primers and 4 μL water. Amplification was performed in a Rotor-Gene Q cycler (Qiagen). The primer sequences with accession numbers or the references^47–49^ used for designing the primers are listed in Supplementary Table S2. Results are expressed as relative values normalized with the expression of the ribosomal protein 13a gene (*RPL13a*) and quantified by the ΔΔCt method. Quantitative differences in gene expression between experimental groups were analyzed using Mann–Whitney, Kruskal–Wallis and Wilcoxon non-parametric tests. Any *p* value of less than 0.05 was considered to be statistically significant.

## Electronic supplementary material

Below is the link to the electronic supplementary material.

Supplementary Information 1.
