# Supplementary material for: Repair of full-thickness articular cartilage defects using IEIK13 self-assembling peptide hydrogel in a non-human primate model
Source: Sci Rep. 2021 Feb 25;11:4560. doi: 10.1038/s41598-021-83208-x (PMC7907267; doi:10.1038/s41598-021-83208-x)
Supplement: Supplementary file 1 — Supplementary Information 1. [file 41598_2021_83208_MOESM1_ESM.pdf]

Repair of full-thickness articular cartilage defects using IEIK13 self-assembling peptide hydrogel in a non-human primate model

Alexandre Dufour[1], Jérôme E. Lafont[1], Marie Buffier[2], Michaël Verset[3], Angéline Cohendet[3], Hugues Contamin[3], Joachim Confais[3], Sharanya Sankar[2], Marika Rioult[2], Emeline Perrier-Groult[1†], Frédéric Mallein-Gerin[1†*]

[1] Laboratory of Tissue Biology and Therapeutic Engineering, CNRS UMR 5305, University Claude Bernard-Lyon 1 and University of Lyon, 7 passage du Vercors, 69367 Lyon cedex 07, France
[2] 3-D Matrix Europe SAS, Caluire, France
[3] Cynbiose, Marcy l'Etoile, Lyon
†Those authors contributed equally to this work
*Corresponding author: f.mallein-gerin@ibcp.fr

Supplementary Figures:

Figure S1: Western-blotting analysis of type I and II collagens in chondrocyte-hydrogel constructs cultured for 21 days in control medium (CTRm) or in medium supplemented with BMP-2, insulin and T3 (BIT), as indicated. Representative western-blots of human (n= 3) and macaque (n =3) samples are shown. Cropped blots are presented here for better clarity and the corresponding uncropped blots are shown in supplemental figures S2 and S3. We compared the amount of proteins produced in equivalent volumes of IEIK13 and fibrin gels (5 μL). On the right, the positions of mature (mature) and unprocessed (pro) collagen chains. Human and macaque chondrocytes show relatively similar protein production in IEIK13 and fibrin gels, in response to BIT.

Figure S2: Uncropped blots corresponding to the western-blots of macaque samples presented in supplemental figure S1 and reproduced here on the left. The areas framed in red on the right correspond to the images shown on the left. On the right, the asterisks indicate non-specific signals and the double-headed arrow indicates that the image has been inverted for the presentation of the cropped blot shown on the left.

Figure S3: Uncropped blots corresponding to the western-blots of human samples presented in supplemental figure S1 and reproduced here. The areas framed in red on the right correspond to the images shown on the left.

Figure S4: Histological images of normal femoral condyle explanted from a non-operated joint. GAG staining and type I and type II collagen immunostaining of coronal sections.

Figure S5: Identification of *in vivo* MSC homing to IEIK13 hydrogel at 82 days after implantation. (A) Hematoxylin and eosin staining of a frontal section of M1 femoral condyle implanted with cell-loaded (CL) or acellular (AC) IEIK13 hydrogel. The dotted lines indicate boundaries between native cartilage (NC) and implants (scale bar: 2 mm). The solid square refers to the image magnification shown in (B). (B) High magnification of the implant protruding in the underlying bone. Note the presence of cells although the hydrogel was originally cell-free when implanted (scale bar: 200 μm). The dotted square delineates a region whose immunostaining is shown in (C). (C) Identification of CD56 and CD146 positive cells by immunostaining on parallel sections (scale bar: 20 μm).

Supplementary Tables:

Table S1: List of primary and secondary antibodies used for western-blotting and immunohistochemistry analyses (AP: alkaline phosphatase, HRP: horseradish peroxidase, Ig: immunoglobulin, IH: immunohistochemistry, WB: western-blotting).

Table S2: Oligonucleotide primers used for the real-time polymerase chain reaction analyses. The source of the databank used for designing the primers is presented as an accession number. When primers have been used in other studies, the references are indicated.

Figure S1

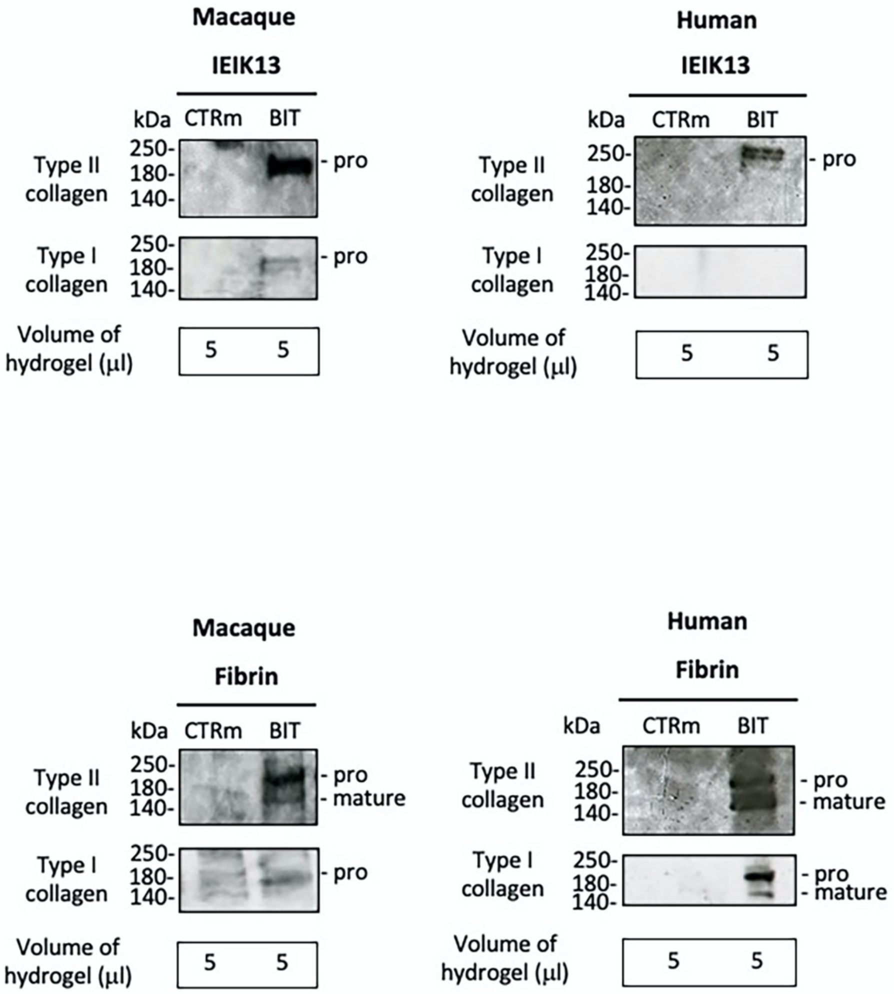
Macaque
IEIK13
kDa
CTRm
BIT
250-
180-
140-
Type II collagen
- pro
Type I collagen
Volume of hydrogel (μl)
5
Human
IEIK13
Macaque
Fibrin
- mature
Human
Fibrin

Figure S2

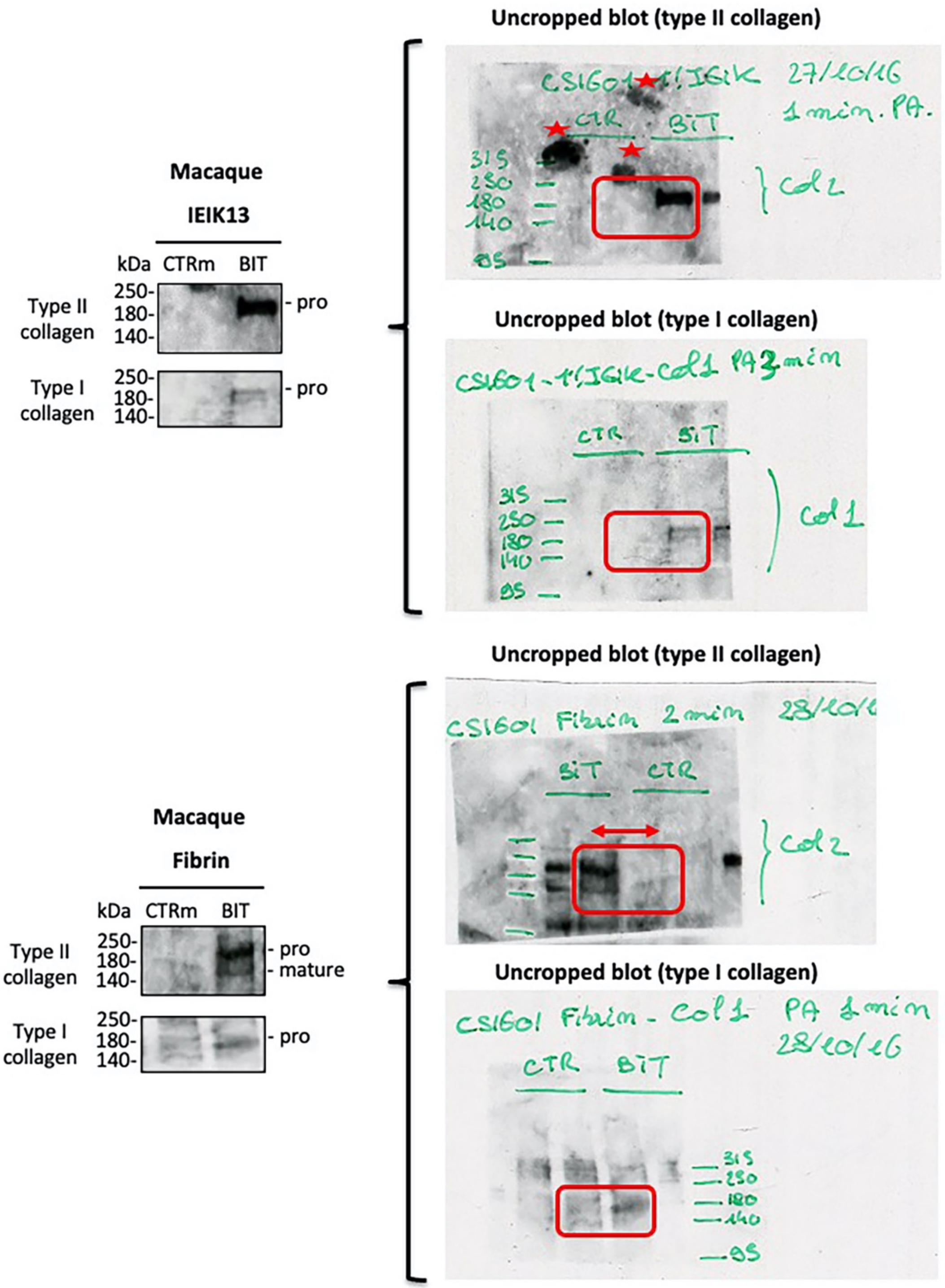
Uncropped blot (type II collagen)
Macaque
IEIK13
kDa CTRm BIT
Type II collagen
250-
180-
140-
- pro
Type I collagen
250-
180-
140-
- pro
Uncropped blot (type I collagen)
Uncropped blot (type II collagen)
Macaque
Fibrin
kDa CTRm BIT
Type II collagen
250-
180-
140-
- pro
- mature
Type I collagen
250-
180-
140-
- pro
Uncropped blot (type I collagen)

Figure S3

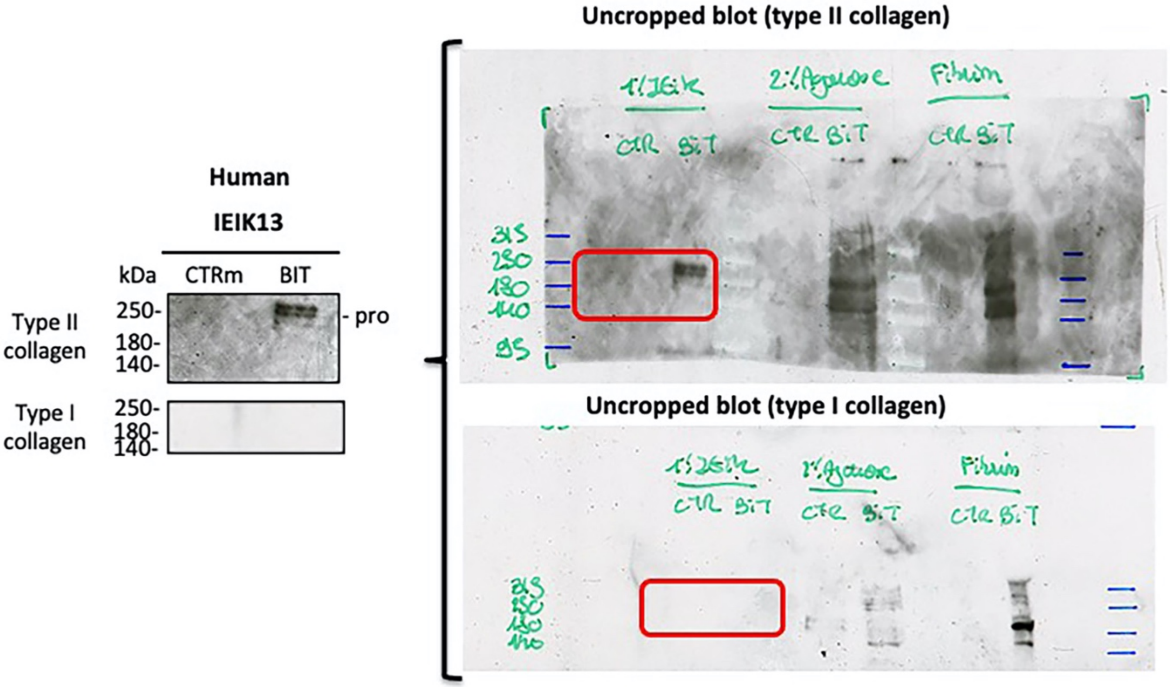
Uncropped blot (type II collagen)
Uncropped blot (type I collagen)
Human
IEIK13
kDa
CTRm
BIT
Type II
collagen
250-
180-
140-
- pro
Type I
collagen
250-
180-
140-

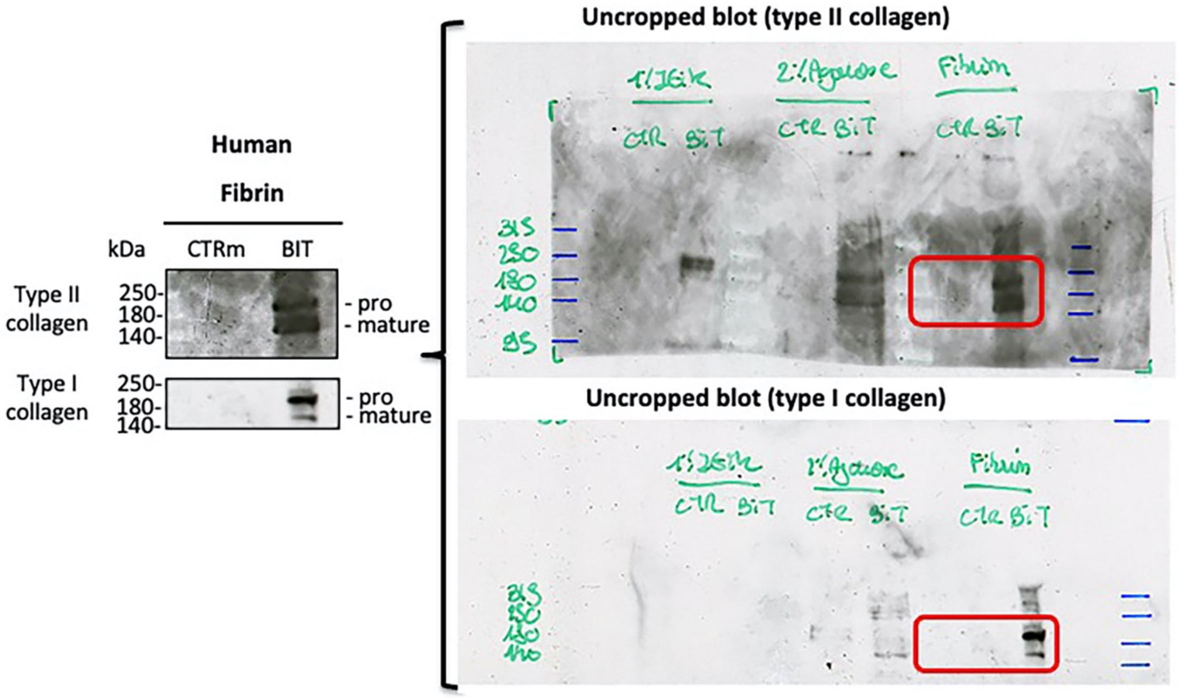
Uncropped blot (type II collagen)
Uncropped blot (type I collagen)
Human
Fibrin
kDa
CTRm
BIT
Type II
collagen
250-
180-
140-
- pro
- mature
Type I
collagen
250-
180-
140-
- pro
- mature

Figure S4

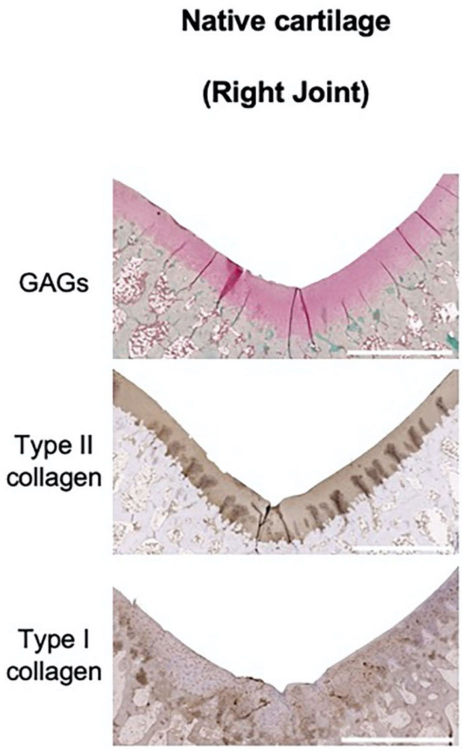
Native cartilage
(Right Joint)
GAGs
Type II collagen
Type I collagen

Figure S5

Right joint - IEIK13
(M1)

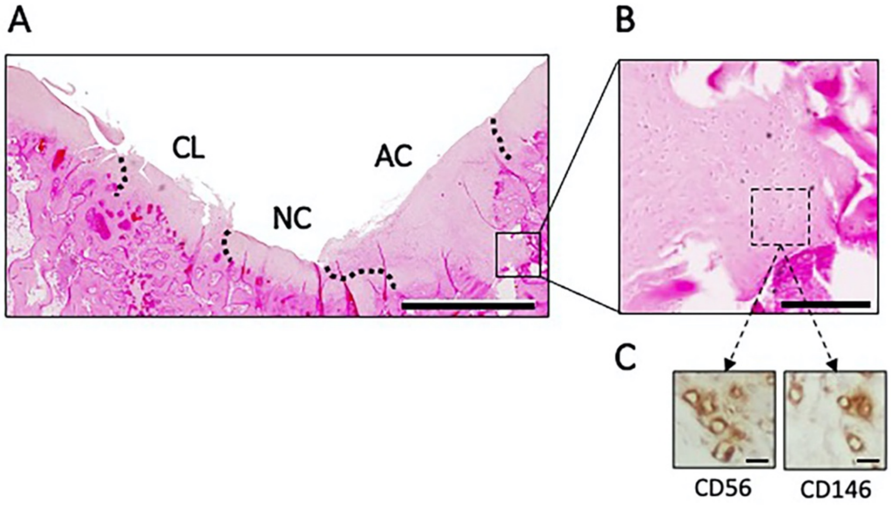

Table S1

Antibodies	Dilution	Source
Polyclonal anti-type I collagen	1:3000 (WB)<br>1:2000 (IH)	Novotec (Ref. 2941)
Polyclonal anti-type II collagen	1:2500 (WB)<br>1:500 (IH)	Novotec (Ref. 370j)
Monoclonal anti-type IX collagen (clone 23-5D1)	1:3000 (WB)	Millipore (Ref. MAB3304)
Monoclonal anti-type X collagen (clone X53)	1:30 (IH)	eBoscience (Ref. 14-9771)
Polyclonal anti-Sox9	1:3000 (WB)	Millipore (Ref. AB5535)
Polyclonal anti-actin	1:2000 (WB)	Sigma (Ref. A2066)
AP-conjugated anti-rabbit IgG	1:3000	Cell Signaling technology (Ref. 05/2016)
HRP-conjugated anti-mouse IgG	1:3000	Cell Signaling Technology (Ref. 11/2010)
HRP-conjugated anti-mouse IgG	Undiluted	Dako (Ref. K4002)
HRP-conjugated anti-rabbit IgG	Undiluted	Dako (Ref. K4002)

Table S2

Gene	*Sequence (5'→3')*	*Species*	*Reference*
RPL13a	Forward: AAAAAGCGGATGGTGGTTC<br>Reverse: CTTCCGGTAGTGGATCTTGG	human<br>monkey	47
COL2A1	Forward: TCCATGTTGCAGAAAACCTTCA<br>Reverse: GGAAGAGTGGAGACTACTGGATTGAC	human<br>monkey	48
ACAN	Forward: TCGAGGACAGCGAGGCC<br>Reverse: TCGAGGGTGTAGCGTGTAGAGA	human<br>monkey	49
COL1A1	Forward: CAGCCGCTTCACCTACAGC<br>Reverse: TTTTGTATTCAATCACTGTCTTGCC	human	49
Col1a1	Forward: CTGGCCTCCCTGGAATGAAG<br>Reverse: CAGGACCAGCATCTCCCTTG	monkey	AF230925.1
